# Stem Cell Therapy and Exercise for Treatment of Intervertebral Disc Degeneration

**DOI:** 10.1155/2021/7982333

**Published:** 2021-10-13

**Authors:** Bo Li, Yunmei Yang, Lan Wang, Guanghua Liu

**Affiliations:** ^1^Department of Geriatrics, The First Affiliated Hospital, College of Medicine, Zhejiang University, Hangzhou, 310003 Zhejiang, China; ^2^Department of Rehabilitation Medicine, Zhongshan Hospital, Fudan University, Shanghai 200032, China

## Abstract

As part of the motor system, intervertebral disc (IVD) is a complicated tissue with multiple components. The degeneration of IVD may result in low back pain (LBP), which strongly impairs quality of life. Various causes are related to the degeneration of IVD, including cell senescence, hydration lost, and inflammation. Stem cells founded in different tissues have attracted the interest of the researchers and clinicians to study the implication of these cells in the treatment for tissue injury and degeneration. In this report, we will review the study of stem cells in the treatment for IVD degeneration. On the other hand, the effect of exercise on IVD degeneration and the relationship between IVD degeneration and musculoskeletal disorders like sarcopenia are discussed.

## 1. Introduction

As one of the major musculoskeletal disorders, low back pain (LBP), with the incidence of 50-80% in adults during lifetime [1], has long been a menace to the public health and the work capacity of the global population [2]. LBP is frequently associated with intervertebral disc degeneration (IDD) and aging. Along with the aging of the population, the economic burden brought by LBP is significantly increasing [3–5]. The total costs of low back pain in the United States exceed $100 billion per year with fewer than 5% of the patients who have an episode of low-back pain account for 75% of the total costs [6]. Patients pay for pain treatment directly and secondary consequences, such as disability, indirectly for this chronic disease. Although low back pain occurs because of multiple etiologies, 40-50% of chronic LBP treated in specialized pain or orthopedic clinics is alleged to be of discogenic origin [7]. Moreover, the same problem is harming the young generation [8]. To reduce the financial cost produced by IDD and its threat to life quality, more efficient diagnostic methodology and treatment are in urgent need [2].

Intervertebral disc (IVD) is a crucial tissue in the motor system containing multiple components with abundant extracellular matrix (ECM) and elaborately regulated vasculature and innervation. Receiving mechanical stimulation continuously during development and aging pose challenges for the health maintenance of the IVD [9] makes the disorder and degeneration of IVD seemly inevitable. IDD occurs with aging brings up gradual structure failure of the disc with inflammation and cell senescence taking parts in it. LBP caused by IDD and aging is a common clinical problem suffered by middle-aged to elderly population, which has a tendency to influence the young generation.

For the treatment of IDD, conservative approaches and surgeries like spinal fusion and total disc replacement are traditional options. With the deepened understanding of stem cells and their power in tissue repair, growing trials assessing the efficiency of stem cells in IDD repair are reported. Meanwhile, strategies should be taken to fix problems like leakage and cell differentiation.

In the end of this review, the relationship between exercise, skeletal muscle defects, and IDD, LBP is discussed. To reduce the damage of IDD and disc aging, the investigation of the etiology and therapy for IDD and disc aging should be strengthened.

## 2. The Structure Overview of IVD

To understand the degeneration and aging of intervertebral disc, its anatomic structure has to be discussed in the first place. The structure of intervertebral disc enabled its function in motor system. In brief, an intervertebral disc is composed of three integrated tissues: a nucleus pulposus (NP), an annulus fibrosus (AF) ring, and two cartilaginous superior and inferior endplates [10]. Being surrounded by annulus fibrosus, the NP is in the central zone of IVD. The NP is a heterogenous structure including sorts of components including cells, collagen fibrils, mineral salts, and water. The water content decreases as aging, which is followed by the metaplasia of the NP. The annulus fibrosus is a complex of concentrically arranged fibrocartilaginous lamellae. It can be divided into inner annulus, which is adjacent to the NP, and outer layer of AF containing concentric lamellae with organized type I collagen fibers. At the end of each vertebral centrum, there is a cartilage end plate, which protects the vertebral centrum from direct pressure [11, 12].

To maintain the metabolism homeostasis and nutrition furnishing of the IVD, blood supply and innervation of intervertebral disc are elaborately regulated.

Branches of spinal artery and young healthy cartilage endplates are observed in the longitudinal ligaments, which are close to the disc. Subsequently, these blood vessels do not present here and leave the NP and adult endplates avascular, which leads to the nutrition exchange of the NP depending on diffusion. Because of the avascular feature of the ventral NP, the concentration of oxygen, some metabolites, and blood is transported by vascular slumps from the AF to the NP, though the lactic acid concentration and hydrostatic pressure are relatively high [13]. Nerves commonly travel along the blood vessels, while some of them also develop without blood vessels [14, 15].

Situating in the main axis of the body, the peculiar segmental and modular structure of the disc provide support and allow flexibility of the body, which means the IVD undertakes a lot of mechanical stimulation. The NP is thought to act as the shock absorber working as a buffer to avoid the direct and drastic mechanical friction and impact on the cartilage and bone [16].

## 3. Degeneration and Aging of IVD

**Figure 1:**
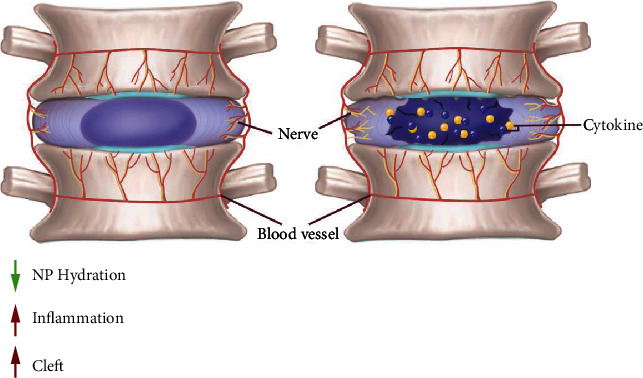
The morphological alteration in degenerative IVD. The schematic diagram shows healthy IVD and degenerative IVD in left and right, respectively. As the degeneration of the IVD, there is hydration loss and appearance of clefts in the bulged NP and fibrotic AF. The inflammation in the IVD intensifies with the increase of sorts of cytokines. Inward innervation which is associated with the pain in IDD is found.

As described above, the constituent of IVD is complicated. The occurrence of aging and degeneration of the IVD is an integral process with all of these components involved. As a matter of fact, for IVD, it is too difficult to draw a line between the process of aging and degeneration. The occurrence of IVD structure defects is intimately connected with aging and degeneration. The two situations have some symptoms in common, but also many differences. Generally, degeneration is frequently found in aging population, aging, however, is not the decisive prerequisite of the IVD degeneration, which also threatens the labor capacity and life quality of middle-aged even young people. The structure failure of the IVD resulted by multiple factors including motor system injury, genetics, and aging, accompanied by the symptoms like neck and back pain, is categorized as degeneration [17]. During the degeneration of the IVD, the components gradually lose their integrity and previous property (Figure 1). With degeneration and aging, the blood supply in the endplates decreases, then the fissure and cleft are seen in the disc and endplates, and the disc becomes more fibrotic and less gel-like. Anatomically, the NP is prompted to bulge as aging, which changes its pressure load and promoted the deterioration of the disc structure. The cells in the AF also turn into more fibrotic. Although there are both the structure destruction of the NP and the AF, it is not clear which one comes first [18]. Cells in AF are partially substituted by fibrotic cells.

Being a dynamic network and part of the regulation of cell behavior, ECM undergoes incessant synthesis and proteinases. It also becomes more disorganized when the disc gets older [19]. The imbalance between synthesis and degradation appears in degenerated disc. More type I and type II collagen are detected in the NP and the outer annulus, respectively. Proteoglycan loses continually. Degraded fragments run off the tissue and the loss of hydration subsequently because of the decreased osmotic pressure [20]. These alterations influence the load bearing of the disc negatively. The tissue microenvironment gets more unendurable with degeneration. The level of cytokines and proinflammatory mediators including IL-1*α*, IL-1*β*, IL-6, and IL-7 increases. The infiltration of immune cells is intensified [21]. Abundant macrophage and mast cell infiltration were observed in patients with LBP. It is also reported that Th17 lymphocyte infiltration increased in degenerated IVDs. These alterations bring about the upregulated degradative enzyme and enhanced collapse of collagens and other ECM [22]. IDD is a main cause of low back pain, in which inflammation plays a role [22, 23]. The inflammatory molecules increased in the response to IDD are shown to exacerbate the degeneration of the disc and cause the discogenic pain [24].

The distribution of blood vessels and nerve fibers in aged and degenerated disc increases. Discogenic pain alarms people about the health of their spine during the progress of the disc degeneration. Behind the pain, it is the disorganization of the nerve growth in the IVD. The evidence from immunohistochemistry staining of clinical samples demonstrated that the ingrowth of sensory nerve is correlated with the chronic back pain. As what has been believed, in healthy disc, the nerve was just found present in the outer layer of the AF. While the nerve fibers were seen in the inner third of the AF even in the NP [25]. Some of the nerve fibers stained in the inner part of disc are accompanied by blood vessel, and some are not. Since there was difference in the marker expression between these nerve fibers, they may function differently in the development chronic back pain. The regulation mechanism of the ingrowth if the nerve fibers and their relationship with discogenic pain still need more research. Except for the extracellular environment, the accumulation of senescent cells is also correlated with aging of the disc [26]. The classic p53-p21 pathway is shown to be involved in this process [27]. NP cells isolated from degenerated disc proliferated slower than those from nondegenerative discs. Accelerated cell senescence was observed in degenerated discs relative to cells in normal discs [28]. It was reported that telomere length decreased while the level of reactive oxygen species (ROS) increased progressively with IDD advancing. The main pathways proposed to mediate the senescence of disc cells include p53-p21-Rb and p16-Rb, Wnt, and mTOR pathways. Except for those classic cell senescence pathways, mitochondrial dysfunction was also found in the AF cells in degenerated discs. It is believed that the cell senescence mediated by these pathways contributes to the negative alteration of the disc environment [29].

## 4. Stem Cell Therapy for IDD

The IVD degeneration and related diseases are usually found by the sensation of the pain, when there have been some physiological or pathological changes in the IVD. Therefore, pain relief through conservative (nonsurgical) means is taken first. That is a time-consuming process with uncertain results because of the cause of the pain is not clearly understood. Additionally, present diagnostic technology cannot tell where the origin of the pain is precisely yet.

When it comes to surgical treatments, there are options mainly including spinal fusion and disc replacement [30]. Spinal fusion is one of the most frequently utilized approaches for diagnosed disc diseases including chronic disc degeneration, spondylosis, and spinal stenosis, of which the lumbar spinal fusion is probably the most operated. With the aim of eliminating movement at degenerated joints to help the stability, spinal fusion also has some negative results such as the adjacent segment degeneration and failure of fusion. To overcome these disadvantages in the treat of IDD, spine surgeons established total disc replacement, in which the disc height was restored without restricting the motion at the operated level. As the optimation of the surgery, more and more options of the material appear. No research with enough short- and long-term results shows that any of the two treatments are superior of the other one. It needs comprehensive consideration to treat a certain disc disease in individual patients.

Certainly, as surgical procedure involving dissection and exposure of the disc, both of the treatments mentioned above have their limitations and complications, for instance, myocardial infarct, pulmonary embolism stroke, and postoperative infections. Moreover, the old people make up a certain percentage of the patients receiving the operations, for whom the recovery is harder because of the declined regenerative capability of the body. More importantly, although the pain may be relieved, the process of degeneration may be delayed, and none of these surgeries is able to reverse or cure the degeneration.

It is because these concerns that the cell therapy drew the attention of scientists and clinicians. Therapy with more efficiency is required. From the totipotent embryonic stem cell to the inducible multipotent stem cell, the possibility of cell fate manipulation motivates people to envision the promising future with stem cell therapy. With the successful application of the stem cell therapy in the treatment for other tissues, we cannot see why it is impossible to deal with IDD with the power of stem cell. Before that, the first thing needs to be thought about is the source of the stem cell.

**Figure 2:**
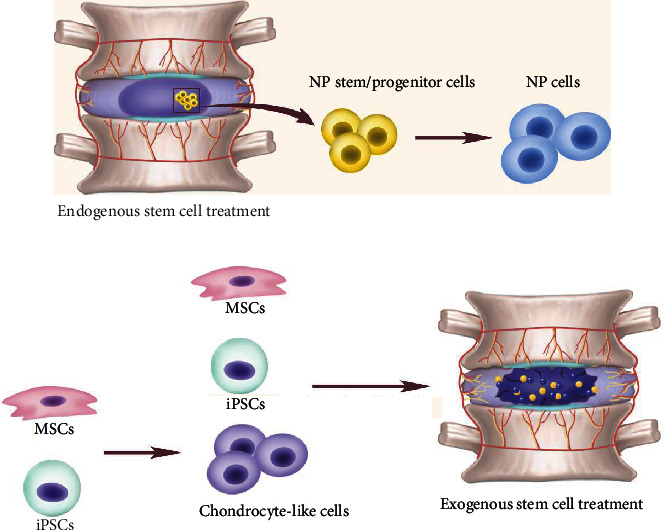
Stem cell treatment for IDD. (a) In the endogenous stem cell treatment, efforts of activating the stem cells in the NP are made to improve the degeneration of the IVD. (b) In the exogenous stem cell treatment, stem cells from other tissues like MSCs and iPSCs are delivered into the disc via intradiscal injection with or without the differentiation before utilization.

Speaking of cell therapy, if there really exists a population in the NP with the ability of self-renewal and differentiation towards NP cells, the stem cells from the NP itself must be the extremely suitable candidate, considering they have been adapted to the IVD microenvironment finely (Figure 2(a)). Thanks to the rapid development of experimental technology, the research of IVD has been more exquisite than ever. The definition and isolation of human IVD stem/progenitor cells have been reported [31–34]. Cell clusters with certain surface markers group in the components of IVD are identified as stem cells with the ability of self-renewal and multipotent differentiation in vitro. Stem/progenitor cells from different parts of the disc, NP, AF, and endplates are isolated and tested for their regenerative capacity. According to the location of the cells, the investigations of stem/progenitor cells in the disc propose that there are NP-derived stem cells (NPSCs), AF-derived stem cells (AFSCs), and CEP-derived stem cells (CESCs) in IVD stem cells which are defined as stem cells if they meet the criteria of mesenchymal stem cells. On the other hand, many researches demonstrated these stem cells may have the same origin or they are cells migrated from other part of the disc [35]. Different groups reported their positive surface markers of the stem/progenitor cells derived from the disc [35–37], with most of the surface markers combinations include CD73, CD90, and CD105, given these markers indicating cell migration and differentiation [38]. Except for these common mesenchymal stem cell markers, tunica intima endothelial kinase (Tie2+) and disialoganglioside (GD2+) are thought to be the markers pinpointing NP progenitor cells [39].  Table 1 listed some of the cell surface markers reported. Even though, the international standard for the disc-derived progenitor/stem cells is still a vacancy waiting to be filled. Thus, more detailed work remains to be done in the digging into the progenitor/stem cells in the disc. From the identification and definition of the stem cells to their application in the treatment for IDD, that is a long way to go [39].

Also, the multipotential differentiation of these cells means it should be carefully induced to make sure the cells differentiated into the right cells that we want, instead of some other cells that make no difference to the treatment or even worse, promote the degeneration [32]. The repair of IDD with disc stem/progenitor cells can be divided into endogenous means, which improves the health of disc by activating the NP stem/progenitor cells in the NP of the degenerated disc, and exogenous way, which depends on the transplantation of disc-derived stem/progenitor cells commonly being preconditioned before the implantation.

Theoretically, tissue get to repair itself after damages happen by a cascade of response including the activation of specific stem cells and recruit of immune cells [40]. However, if the accumulative injury or stress is much more than the tissue is able to repair, the unsolved leisure may turn into degeneration or aging. The progenitor/stem cells located in the NP itself were found to decrease markedly in patients with aged and degenerated disc [36]. Ex vivo study also observed that the NP progenitor cells isolated from patients whose disc undergoes degeneration of different severity show declined cell properties like decreased proliferation, colony-formation capacity, migration, chondrogenic ability, and aggravated senescence. In the human NP samples from degenerated disc, the mesenchymal stem cell marker expression is higher in the young while lower in the old ones [41]. Similar results were found in study with rabbits as animal model [42]. The influence on the resident stem cells of the disc of aging and degeneration to some extent accounts for the impaired function and regeneration of the disc after injury and overload. Therefore, the activation of the resident stem cells in the disc may help to arrest or even reverse the collapse of it. Otherwise, if its own stem cells are too difficult to mobilize, the transplantation of exogenous stem cells from healthy NP and AF is optional.

It is more feasible for the researchers nowadays to examine the regeneration-facilitating ability of disc-derived stem/progenitor cells in vivo through animal models or clinical trials. Still, mice as model are too small for cell isolation, tissue manipulation, and quantitative analysis. Other models like rat, rabbit, or pig are alternative. Some encouraging results about the application of the disc-derived progenitor/stem cells in the treatment for IDD have been shown. In a study by rabbit model, they found better recovery results in NP stem cell transplantation than that of the whole NP cells [43]. Considering the disc may restore through its endogenous stem cells, investigators contrived to activate stem cells in the degenerated disc in lieu of the transplantation of exogenous cells. In an in vivo study, simvastatin was used to enhance the differentiation of nucleus pulposus mesenchymal stem cells into nucleus pulposus because of its ability of promoting expression of hypoxia inducible factor [44]. Stem cell therapy application in human is increasing with autologous MSCs as the main choice. Injection of autologous MSCs and other stem cells in IDD back pain treatment has been reported around the world. In a MSC injection for treatment of lumbar spinal stenosis with 2 clinical cases, enhanced stability and reduction of air in the IVD of the patients were reported 2 years after the operation [45]. Except for MSCs, autologous NP cells were also used in some cases. In another clinic report, the NP cells isolated from patients suffering from IDD and LBP were cocultured with MSCs and then transplanted to the IVDs after fusion surgery. Pain relief was reported from all the patients 3 years after the transplantation [46]. Although pain score decrease and hydration increase were reported in these clinical cases, it was hard to get a higher disc height index [47]. More clinical studies with longer follow-up period and larger patient amount are still needed to prove the safety and effects of stem cell therapy.

Behind the bright side, many obstacles are unignorable for the utilization of the disc-derived progenitor/stem cells. Not all the trials of the endogenous repair are successful [48]. In the harsh microenvironment of the disc, every step should be right for the cells to survive and function as the expectation. Even the progenitor cells in the NP have been accustomed to the microenvironment, and compression and hypoxia may still induce their apoptosis [49]. After survival, the hypoxic environment is a challenge for other cell behaviors of the stem cells. Proper hypoxic (2% O_2_) may facilitate the migration of the cells, but excessive hypoxic (less then 1% O_2_) would repress the migration [50, 51]. Even they finally migrate to the desired spot in the disc, the differentiation into functional cells the production of related ECM is still not a certainty. Thus, being thrilled about the progress the field has made, besides, a comprehensive understanding of the microenvironment of the disc and the mechanism of repair remains to be explored.

Due to many details about the development and repair of IDD is still scarce, it is difficult to obtain the young and healthy NP cells for degeneration repair. Therefore, pluripotent, multipotent stem cells, which can be induced to differentiation towards a certain cell type, and adult stem cells that can be isolated from the patients like mesenchymal stem cells are becoming ideal and promising options when biomedicine is applied in IVD regeneration (Figure 2(b)). These stem cells can be acquired from multiple tissues including bone marrow, adipose tissue, synovium, and embryonic stem cells (ESC).

The application of mesenchymal stromal/stem cells (MSCs) in the repair and regeneration of injured tissues has been a hotspot for a long time [52–54]. Comparing with the resident progenitor/stem cells of the disc, the MSCs from other tissues own many specific advantages. Most importantly, they can be acquired from multiple tissues and applied widely [55, 56]. The trials examining the efficacy of the MSCs in the repair of injury have been conducted in manifold tissues including but not limited to lung [57], liver [58], and bone [59]. Beyond that, the trend of its utilization in tissue repair and regeneration keeps growing, not receding [60, 61].

In the strategies of repairing disc injury by using MSCs, the cells delivered into the disc may be treated in different ways [62]. The cells are in differentiated or undifferentiated state during injection, keeping the cells undifferentiated with the benefit of more cellular viability and potential and shortcomings like unexpected differentiated linages which affect the treatment. While the preconditioning of the cells before usage leaving a vast space for the researchers and clinicians to transform the cells and make them more adaptive for the new growth environment. Various modalities manipulate the differentiation of the MSCs by physical and biological stimulation. Physically, mechanical stimulation or hypoxia treatment was used on the cells. Biologically, cells are stimulated by factors like transforming growth factor- (TGF-) *β*, insulin-like growth factor- (IGF-) 1 [63, 64], or precultured with NP cells to improve the function of the cells by cell-cell contact [65, 66]. Exposure of the cells in simvastatin [44], lithium [67], and platelet-rich plasma [68] is also shown to improve the survival and proliferation efficacy of kinds of stem cells. The differentiation of MSCs derived from human tissues into NP-like cells has been reported [69, 70].  Table 2 gave some examples of the modification of the cells in treatment.

In addition to the source of the stem cells, the delivery and carry of the cells are also of concern. The therapeutic cells have to be injected because of the lack of vasculature in IVD. The environment of IVD is tough since the space is restrained and hypoxic. The stem cells injected into it do not survive readily, let alone proliferating and producing the matrix that the NP needs as imagination [71]. Besides, the cell leakage is an unignorable problem [72], because of which the hydrogel utilized in IVD injection is hoped to be solidified after implantation. The stem cells leaked through the needle tracks may not only lead to the diminished repair effect but also be a potential risk of spinal stenosis, osteophyte formation, and tumor growth. That is why cell carrier with excellent properties is in need [73, 74]. Research injecting MSCs in the disc of rabbit IDD model found osteophytes out of the nucleus 9 weeks after the injection. The GFP labelled MSCs, which were not in the nucleus, were found among the osteophyte-forming cells. As a highly loaded tissue, material migrated from the disc may hinder the normal function of nerves around the disc and leads to complications including paralysis. For clinical safety, the observation of the injected mixture after treatment should be taken into account. Stable retention of the material and cells injected should be detected through noninvasive assessments such as radiopaque contrast agents and nanoparticles.

No matter where the stem cells are derived or what the cell carriages are, the final aim of the cell therapy is to retard or even reverse the degeneration of the disc from all angles. It means to recover or get close to the normal situation of the disc by replenishing the ECM through functional NP cells, to restitute the mechanical bearing and support for motion of the spine, and to make the disc regain the homeostasis of metabolism. Since the research about IVD has established more in-depth comprehension, the attempt at the clinical application of biomedical therapy including stem cell therapy has not stopped [73]. The massive potential of stem cell therapy in treatment for IDD degeneration and IVD aging is still waited to be exploited.

## 5. The Effect of Exercise on IVD Degeneration

Except for the natural aging, the degeneration of disc and the formation of many disc diseases have intimate relationship with individual life habits like exercise, sleep, work, and smoking. Among these factors, exercise and sedentary may influence the health situation of the disc heavily. As the mode of production changes, people work in fore bending gesture more than they ever did. The convenience of modern society normalizes sedentary lifestyle. All the alterations pose a threat to the health of the disc, which may accelerate the degeneration that happens naturally with aging.

Continuous exercise with proper intensity benefits the body from all sides. The influence of exercise on physiology and pathology has long been discussed [75–77]. Mechanical stimulation of stem cells is a common approach in tissue engineering. Tissues of the body, especially those in the motor system, accept mechanical stimulation all the time. Stem cell therapy with mechanically stimulated stem cells and appropriate scaffold is used to help recovering the function of tissues including skeletal muscle, cartilage, and tendon after degeneration or injury [78, 79]. Researches on patients of developing osteoarthritis (OA) and animal models show that moderate physical activity has positive influence on the cartilage integrity. While overload situations like obesity would be risk factors [80, 81]. It is the crucial regulatory function of exercise that leads the researchers to test the effects of mechanical stimulation of stem cells on therapy.

How exercise would affect the degeneration and aging of IVD and LBP is also of great concern. The conclusion of the problem whether exercise like running is beneficial to the improvement of IDD or LBP is still suspended [82, 83]. Although studies with rat as animal model show that running training increased the cell number and ECM expression in NP and AF and alleviated the pain of degenerated IVD [84, 85]. A 6-month randomized control trial involving 40 patients found no significant difference between the control group and the exercising counterparts on the symptom of chronic low back pain [86]. While another research argued that human IVD responds positively to running exercise [87]. It may be difficult to define the effect of exercise on IDD and disc aging, but long sedentary work and disuse of motor system are not helpful for the control of IVD disorders.

Since mechanical stimulation including exercise related to the health of spine, musculoskeletal disorders would interfere the normality of the disc. Except for some congenital diseases, the decline of the motor system resulted by decreased bone and muscle as well as the accumulative senescent cells in these tissues commonly occurs with aging. These exogenous factors from the musculoskeletal system could threaten the health of spine.

## 6. Sarcopenia and IVD Degeneration

Sarcopenia appearing with aging brings up the symptoms like progressive skeletal muscle loss, impaired strength, and function of muscle [88]. The research paying attention on the association between sarcopenia and disc degeneration is in sparsity. Clinically, they question the relationship between sarcopenia and low back pain [89]. A meta-analysis comprising 1953 participants shows that sarcopenia accounts for approximately a quarter of the population who suffer from lumbar degenerative spine diseases [90]. However, there is not yet quantitative analysis that demonstrates the causal relationship between sarcopenia and back pain or disc degeneration, and more data and statistical analysis are needed for improved diagnoses and treatment. Being different from sarcopenia, back muscle degeneration, with muscle functional failure of the back specifically, is also related to low back pain [91, 92]. Comparative study reported that back muscle degeneration seemly has more association with back pain than sarcopenia does [93]. The degeneration of both disc and back muscle increases with age [94]. During the degeneration of muscle, it gets small in size, and some of the space is infiltrated by fat tissue. The paraspinal and psoas muscle are considered to be essential to stabilize the spine. The degeneration of muscle would decrease the force it provides to support the spine. Even though back muscle degeneration is reported to be associated with spinal symptoms like low back pain, spinal kyphosis, and spinal stenosis, given the complicated etiology of IDD, it is still hard to conclude that back muscle degeneration is a main reason of it [95].

## 7. Conclusion

As our lifestyle changes and the global population ages, the obstacles brought by degenerative diseases will draw increasing attention. Here, we discussed the study about the mechanism of the degeneration and aging of the IVD, the exploration researchers have done to extend the possibility of treatment, and finally, the relationship between exercise and IVD degeneration and aging, one of the major musculoskeletal disorders. Except for the existing progress, some key questions require urgent answers. As mentioned above, in the attempt to treat IDD with stem cells, the delivery and solidification of the mixture of cells and hydrogel (or other material) are crucial to the treatment result and leakage-related adverse effect control. For endogenous treatment by activating the stem/progenitor cells in the disc, no consensus has been reached on a certain type of cell population. With the emergence of new technologies especially single-cell RNA-seq, updated information about stem cells in the disc can be expected. For exogenous treatment with stem cells from other tissues, the differentiation of these cells and safety issue is of concern. Exercise with proper protocol and intensity may help to decelerate the degeneration of the IVD. However, more clinical statistics of the patients are needed to draw a conclusion. Musculoskeletal disorders like sarcopenia are probably associated with development and diagnosis of IDD. However, experimental evidence is required to make a solid statement. The combination of clinical questionnaire and diagnose and basic research may facilitate the progress of revealing the truth.

## Figures and Tables

**Table 1:** The cell surface markers reported.

Stem cell type	Cell surface markers	Reference
Mouse and human NP progenitors	Tie2+ and GD2+	Ref. [36]
Rat NPMSCs	CD73+, CD90+, and CD105+	Ref. [37]
Human NPSCs	CD73+, CD90+, CD105+, CD34-, and CD45-	Ref. [35]
Human annulus progenitors	CD29+, CD44+, CD105+, and Cd14+	Ref. [38]

**Table 2:** Some examples of the modification of the cells in treatment.

Cell type	Modification	Reference
NP-derived mesenchymal stem cell (NPMSC)	Cell pretreatment by simvastatin	Ref. [44]
Adipose-derived stem cell (ADSC)	Cell injection with microsphere loaded with dexamethasone and growth factor	Ref. [63]
Bone marrow-derived mesenchymal stem cell (BMSC)	Cell pretreatment by TGF-1	Ref. [64]
Mesenchymal stem cell (MSC)	Coculture with nucleus pulposus cells	Ref. [65]
NP-differentiated mesenchymal stem cell	Hypoxic culture environment	Ref. [66]
Adipose-derived stem cell (ADSC)	Cell pretreatment by LiCl	Ref. [67]
Mesenchymal stem cell	Cell pretreatment by hyaluronic acid (HA) and platelet-rich plasma (PRP)	Ref. [68]
